# Supplementary material for: The Characteristics of Multilocus Sequence Typing, Virulence Genes and Drug Resistance of Klebsiella pneumoniae Isolated from Cattle in Northern Jiangsu, China
Source: Animals (Basel). 2022 Sep 30;12(19):2627. doi: 10.3390/ani12192627 (PMC9558562; doi:10.3390/ani12192627)
Supplement: Supplementary file 1 [file animals-12-02627-s001.zip › animals-1926474-supplementary.pdf]

Table S1. Primers used for the PCR experiment.

Genes	Sequence（5′ – 3′）	Products（bp）
gapA	F：TGAAATATGACTCCACTCACGG<br>R：CTTCAGAAGCGGCTTTGATGGCTT	663
infB	F：CTCGCTGCTGGACTATATTCG<br>R：CGCTTTCAGCTCAAGAACTTC	463
mdh	F：CCCAACTCGCTTCAGGTTCAG<br>R：CCGTTTTTCCCCAGCAGCAG	757
pgi	F：GAGAAAAACCTGCCTGTACTGCTGGC<br>R：CGCGCCACGCTTTATAGCGGTTAAT	718
phoE	F：ACCTACCGCAACACCGACTTCTTCGG<br>R：TGATCAGAACTGGTAGGTGAT	603
rpoB	F：GGCGAAATGGCWGAGAACCA<br>R：GAGTCTTCGAAGTTGTAACC	1076
tonB	F：CTTTATACCTCGGTACATCAGGTT<br>R：ATTCGCCGGCTGRGCRGAGAG	540
rmpA	F：ACTGGGCTACCTCTGCTTCA<br>R：CTTGCATGAGCCATCTTTCA	516
wcaG	F：GGTTGGKTCAGCAATCGTA<br>R：ACTATTCCGCCAACTTTTGC	169
allS	F：CCGTTAGGCAATCCAGAC<br>R：TCTGATTTATCCCACATT	1090
kfuBC	F：GAAGTGACGCTGTTTCTGGC<br>R：TTTCGTGTGGCCAGTGACTC	797
ybtA	F：ATGACGGAGTCACCGCAAAC<br>R：TTACATCACGCGTTTAAAGG	960
iucB	F：ATGTCTAAGGCAAACATCGT<br>R：TTACAGACCGACCTCCGTGA	948
iroNB	F：GGCTACTGATACTTGACTATTC<br>R：CAGGATACAATAGCCCATAG	992

fimH	F：GCTCTGGCCGATACCACCACGG<br>R：GCGAAGTAACGTGCCTGGAACGG	423
ureA	F：GCTGACTTAAGAGAACGTTATG<br>R：GATCATGGCGCTACCTCA	337
uge	F：GATCATCCGGTCTCCCTGTA<br>R：TCTTCACGCCTTCCTTCACT	534
wabG	F：CGGACTGGCAGATCCATATC<br>R：ACCATCGGCCATTTGATAGA	683
